# Effect of Adjuvants on Oxytetracycline Uptake upon Foliar Application in Citrus

**DOI:** 10.3390/antibiotics9100677

**Published:** 2020-10-06

**Authors:** Nabil Killiny, Faraj Hijaz, Pedro Gonzalez-Blanco, Shelley E. Jones, Myrtho O. Pierre, Christopher I. Vincent

**Affiliations:** 1 Department of Plant Pathology, Citrus Research and Education Center, IFAS, University of Florida, Lake Alfred, FL 33850, USA; fhijaz@ufl.edu (F.H.); pcgo@ufl.edu (P.G.-B.); shjones@ufl.edu (S.E.J.); 2 Department of Horticultural Sciences, Citrus Research and Education Center, IFAS, University of Florida, Lake Alfred, FL 33850, USA; mypierre@ufl.edu (M.O.P.); civince@ufl.edu (C.I.V.)

**Keywords:** oxytetracycline, adjuvant, foliar spray, cuticle, laser, citrus, Huanglongbing

## Abstract

Recently in Florida, foliar treatments using products with the antibiotics oxytetracycline and streptomycin have been approved for the treatment of citrus Huanglongbing (HLB), which is caused by the putative bacterial pathogen ‘*Candidatus* Liberibacter asiaticus’. Herein, we assessed the levels of oxytetracycline and ‘*Ca*. L. asiaticus’ titers in citrus trees upon foliar applications with and without a variety of commercial penetrant adjuvants and upon trunk injection. The level of oxytetracycline in citrus leaves was measured using an oxytetracycline ELISA kit and ‘*Ca*. L. asiaticus’ titer was measured using quantitative PCR. Low levels of oxytetracycline were taken up by citrus leaves after foliar sprays of oxytetracycline in water. Addition of various adjuvants to the oxytetracycline solution showed minimal effects on its uptake by citrus leaves. The level of oxytetracycline in leaves from trunk-injected trees was higher than those treated with all foliar applications. The titer of ‘*Ca*. L. asiaticus’ in the midrib of leaves from trees receiving oxytetracycline by foliar application was not affected after four days and thirty days of application, whereas the titer was significantly reduced in oxytetracycline-injected trees thirty days after treatment. Investigation of citrus leaves using microscopy showed that they are covered by a thick lipidized cuticle. Perforation of citrus leaf cuticle with a laser significantly increased the uptake of oxytetracycline, decreasing the titer of ‘*Ca*. L. asiaticus’ in citrus leaves upon foliar application. Taken together, our findings indicate that trunk injection is more efficient than foliar spray even after the use of adjuvants. Our conclusion could help in setting useful recommendations for the application of oxytetracycline in citrus to improve tree health, minimize the amount of applied antibiotic, reduce environmental exposure, and limit off-target effects.

## 1. Introduction

Citrus greening disease, also known as Huanglongbing (HLB), is currently considered the most harmful citrus disease in the US and much of the world, causing trees to decline rapidly, with accompanying loss of production [1]. In the Americas, HLB is caused by a phloem-limited bacterium ‘*Candidatus* Liberibacter asiaticus’, and it is transmitted by the Asian citrus psyllid, *Diaphorina citri* Kuwayama [2]. To date, no cure for HLB has been found, and the disease management strategies rely heavily on the use of insecticides to control the insect vector. Several other control methods including thermotherapy and tree removal were proposed for the management of the HLB disease. However, these methods were not effective enough in the field [3].

The use of antibiotics was approved in 2016 for the treatment of HLB in Florida due to the significant economic losses throughout the citrus industry. Antibiotics such as oxytetracycline and streptomycin have been successfully used for treatment of several plant pathogens for more than seventy years [4]. For example, fire blight of apple and pear caused by *Erwinia amylovora*, and bacterial spot disease of peach and nectarine caused by *Xanthomonas arboricola* have both been controlled by oxytetracycline [5]. Oxytetracycline has also been used for the management of *Pseudomonas* spp. and *Xanthomonas* spp. in some vegetable crops, as well as for phytoplasmas that induce the lethal yellows disease in elm and coconut palm trees [5].

Early studies on the use of antibiotics for the control of HLB disease were initiated in the 1970s after it was proposed to be caused by a microbial plant pathogen [3]. Early studies demonstrated the efficacy of tetracycline trunk injections to significantly reduce HLB symptoms in infected citrus trees [6,7,8,9]. These studies also showed that foliar sprays were less effective than trunk injections [10]. Another study showed that achromycin was also effective against HLB when injected into the trunk of infected plants [10]. Unfortunately, studies regarding the use of antibiotics for HLB control were discontinued in the beginning of 1980s [11]. Recently, the use of antibiotics was again proposed for HLB control due to the substantial damage caused by the disease since its arrival in Florida in 2004. The results of a recent study showed that that penicillin, ampicillin, rifampicin, carbenicillin, cefalexin, and sulfadimethoxine were all effective against ‘*Ca*. L. asiaticus’ [11]. Other studies reported that streptomycin and penicillin attenuated ‘*Ca*. L. asiaticus’ titer and HLB symptoms [12,13]. Trunk injections of streptomycin, oxytetracycline hydrochloride, and penicillin were shown to be effective against HLB [14].

The effectiveness of agrochemicals depends on their ability to reach their target sites [15]. For example, to be effective, herbicides should translocate to the foliar meristem, while insecticides should reach the phloem and xylem tissues to affect phloem- and xylem-feeding insects [15]. Similarly, the efficiency of the antimicrobial compounds in planta depends on the ability of antimicrobials to reach their site of action. Spiroplasmas, for example, are sensitive to many types of antibiotics; however, only oxytetracycline is effective against Spiroplasmas in planta, indicating that it is highly translocated in citrus trees and it can reach the phloem at high concentrations [16]. 

In our previous study, we examined the uptake and translocation of oxytetracycline and streptomycin in citrus plants upon stem and root delivery [17]. Our results showed both antibiotics were detected in phloem (bark tissue), xylem (inner stem tissue), leaf, and roots after stem injection and root drenching applications. The presence of oxytetracycline and streptomycin in the phloem tissues, where the ‘*Ca*. L. asiaticus’ resides, indicated that they could be effective against this plant pathogen. After incubation of the roots in antibiotic solutions, the highest level of antibiotics was detected in the root, followed by the xylem [17]. On the other hand, high levels of both oxytetracycline and streptomycin were detected in the canopy after delivery by stem injection, and only trace amounts were detected in the roots, indicating limited translocation downward (from shoot to root) of oxytetracycline and streptomycin [17]. Our results agreed with earlier findings showing high level of antibiotics in the canopy following trunk injection, and high levels detected in root after root drench [13,18,19,20]. Oxytetracycline and streptomycin were detected in treated-plants thirty-five days after treatment, indicating good persistence of these antibiotics in citrus [17].

Recently, we used fluorescence-labeled (FL) penicillin to trace penicillin movement in citrus plants [21]. FL-penicillin was observed in the phloem and xylem below and above the application site. The levels of FL-penicillin observed above the application site was higher than below, and the levels in the phloem were lower than in the xylem [21]. In addition, girdling of the bark did not prevent the translocation of FL-penicillin, indicating that it was primarily translocated via the xylem first, then diffused horizontally to phloem [21]. Furthermore, FL-penicillin was detected in the gut of *D. citri* fed on treated plants, indicating that FL-penicillin was present in the phloem and/or xylem of treated plants.

Foliar spray is a common method for applying pesticides, herbicides, and nutrients to crops. However, as little as 0.4% of the applied insecticide may reach its target and the rest of the applied material is deposited in the environment [22]. Soil drenching is utilized as an alternative to spraying in order to minimize chemical loss [23]. In this method, chemicals are applied to the soil in excess amount around the tree in order to be taken up by the roots [23]. Soil drenching is used for many insecticides including neonicotinoids, which are used to control *D. citri* [24]. However, only a small fraction of the chemical is taken up by plants from the root zone, and the rest may persist in the soil long-term, which could have adverse environmental effects. Due to these risks for the environment and for human health, trunk injection was developed as an alternative method [24]. Trunk injections are widely used in urban areas where sprays and soil drench techniques are impractical, prohibited, or restricted [24]. Tree trunk injection offers several advantages over foliar spray and soil drench methods: a) it provides an accurate dose; b) there is no soil deposition, drift loss, or abiotic or biotic degradation; c) it reduces non-target impacts and user exposure; d) it can control borer insects that feed under the bark, e) longer persistence of action means fewer treatments per year; f) and fewer applications may make trunk injections a more affordable investment [24]. 

To perform their desired biological function, agrochemical applied using foliar spray should be transferred from the leaf surface into the plant tissues. Consequently, foliar agrochemicals need to overcome the first barrier of the leaf, the cuticle. The plant cuticle is composed of water-repellent waxes (long-chain hydrocarbons, fatty acids, alcohols, and esters), less water-repellent cutin (esterified fatty acids), and pectin which provide pathways for water soluble compounds [25,26]. Consequently, adjuvants are mixed with agrochemicals to enhance their performance [27]. Activator adjuvants affect the physical and chemical properties of the spray solution, which in turn modify the wetting, spreading, retention, or penetration of the foliar spray [27].

Oxytetracycline and streptomycin have been approved in Florida by the Environmental Protection Agency (EPA) for the treatment of ‘*Ca*. L. asiaticus’-infected trees using foliar application since 2016 [28]. Here, we hypothesize that only trace amounts of antibiotics are taken up by citrus trees upon foliar spray application, and the use of activator adjuvants could enhance their uptake by citrus trees. Consequently, we tested the levels of oxytetracycline in citrus trees upon foliar application with and without adjuvants and compared the detected levels to those found after trunk injections. 

## 2. Results

### 2.1. Adding Adjuvants to Oxytetracycline Does Not Increase Its Uptake by Citrus Leaves upon Foliar Application (Field Study)

**Figure 1:**
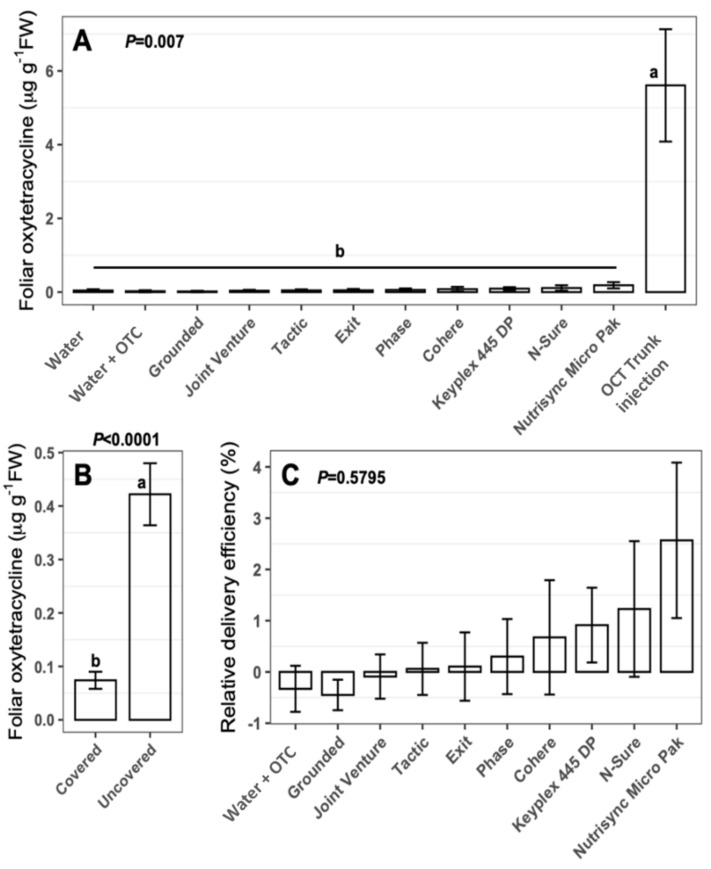
The effect of adjuvants on the uptake of oxytetracycline upon foliar application in comparison to truck injection. (**A**) The level of oxytetracycline (µg·g^−1^ FW) in covered leaves from control, foliar-treated, and trunk injected ‘Hamlin’ sweet orange trees was assessed four days after treatment (*n = 12*). See material and methods for more details. Treatments with different letters are significantly different using Tukey’s test. (**B**) The level of oxytetracycline (µg·g^−1^ FW) in covered and uncovered leaves from foliar-treated ‘Hamlin’ citrus trees four days after treatment. The levels of oxytetracycline in leaves collected from all foliar-treated trees except the control (water) were pooled together (*n = 144*) and analyzed using *t*-test. Treatments with different letters are statistically significantly different. (**C**) Relative efficiency of adjuvants in the uptake the oxytetracycline. The relative efficiency of systemic delivery of foliar application treatments was calculated by considering injection to represent 100%, and the control (water-oxytetracycline) to represent 0%. Statistically significant differences were evaluated at *p* < 0.05.

A field experiment using infected citrus trees was carried out to test the effects of various adjuvants on the uptake of oxytetracycline upon foliar spray. The level of oxytetracycline in leaves from trees that were sprayed with oxytetracycline solution was very low and it was not significantly different (using Tukey’s and Dunnett’s tests) from those treated with water alone (Figure 1A). All other adjuvant treatments were not different from the water treatment (Figure 1A). On the other hand, the level of oxytetracycline in trunk-injected trees (used as control) was significantly higher than all foliar treatments (Figure 1A). In order to study the translocation of oxytetracycline upon foliar treatment, some branches were covered with plastic bags to avoid direct contact with the treatment. The level of oxytetracycline in leaves from covered branches was significantly lower than leaves from uncovered branches (Figure 1B). The systemic movement of applied oxytetracycline was about 20% of leaves receiving direct foliar application (Figure 1B). The previous results indicated that a very low amount of oxytetracycline was taken up by citrus leaves after foliar application even after the addition of adjuvants, and subsequently the translocation from uncovered branches to covered branches was poor.

The efficiency of delivery of the foliar treatments was calculated on the covered sample by considering injection to represent 100%, and the control (water-oxytetracycline) to represent 0%. Using this standardization, we calculated the effectiveness of each adjuvant (Figure 1C). The most efficient adjuvant was Nutrisyn Micro Pak and it had a mean of 2.5% systemic delivery efficiency (Figure 1C). However, ANOVA analysis showed no difference among all foliar treatments (Figure 1C).

### 2.2. Foliar Treatments of Oxytetracycline Did Not Reduce the ‘Ca. L. asiaticus’ Titer in Citrus Trees

**Figure 2:**
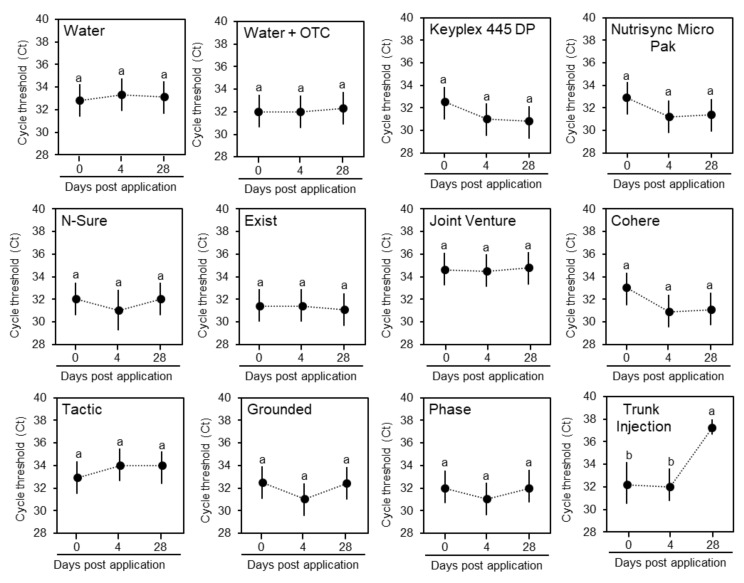
The effect of foliar application with oxytetracycline combined with adjuvants on the ‘*Ca*. L. asiaticus’ titers in citrus leaves in comparison to truck injection. Data are shown as cycle threshold values (Ct) in leaves at zero, four, and thirty days after treatment with water, and oxytetracycline foliar spray, and trunk injection (*n* = 12). The lower Ct value indicates higher titer of ‘Ca. L. asiaticus’. Negative control was performed with DNA extracted from healthy trees while positive control was DNA extracted from infected trees kept in the greenhouse. Different letters indicate statistically significant differences using ANOVA followed by Tukey’s test.

We combined the previous experiment with assessment of ‘*Ca*. L. asiaticus’ titers a day before treatments, 4 d after treatments, and 30 d after treatments. The level of ‘*Ca*. L. asiaticus’ titer was determined in the midrib of the leaves (Figure 2). The ‘*Ca*. L. asiaticus’ titer was significantly reduced in oxytetracycline-injected trees (Figure 2). The cycle threshold (Ct) in oxytetracycline-injected trees increased from 32 to 37 indicating reduction in the bacterial titer and that the oxytetracycline reached the minimum inhibitory level (Figure 2). On the other hand, no differences were observed on the Ct values in all foliar treatments indicating that none of them reached the minimum inhibitory level (Figure 2). This result was in agreement with the high level of oxytetracycline in midribs obtained from trunk-injected trees and the very little amount found in midribs obtained from foliar applied trees.

### 2.3. Incubation of Citrus Leaves in Oxytetracycline Solution Enhances the Uptake

**Figure 3:**
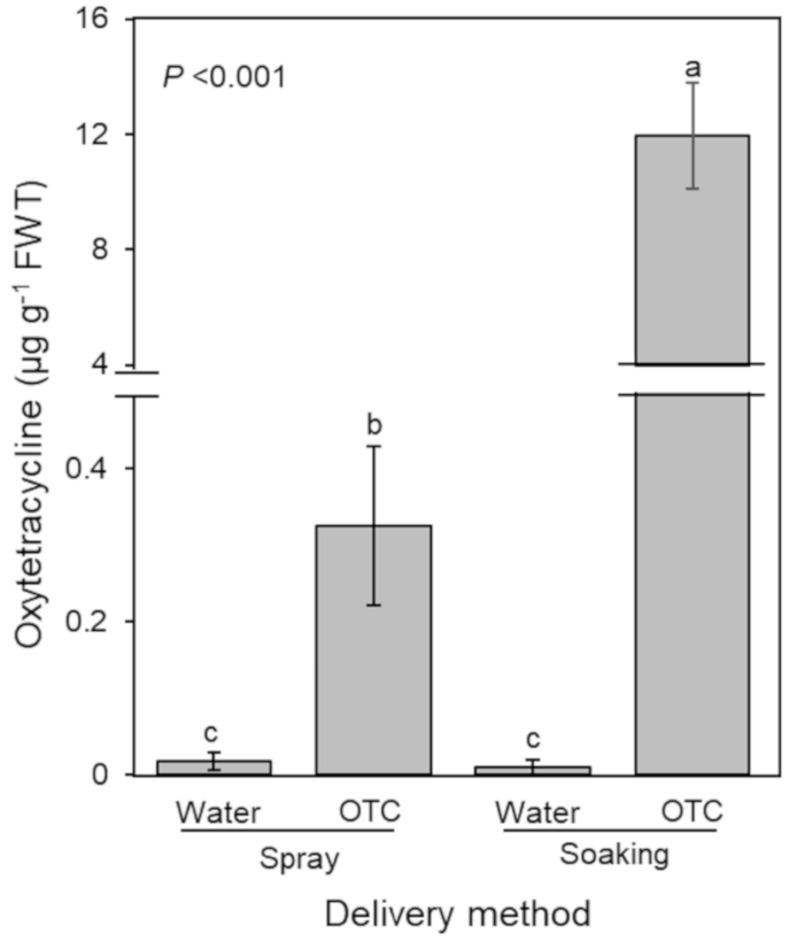
The level of oxytetracycline in citrus leaves after attached leaves were incubated in or sprayed with oxytetracycline solution (200 µg·mL^−1^). The level of oxytetracycline (µg·g^−1^FW) in leaves was assessed four days after treatment (*n = 5*). In the incubation treatment, intact leaves (not detached from tree) were incubated in 8 mL of 200 µg·mL^−1^ oxytetracycline solution in a bag for 3 d. In the foliar treatment leaves were sprayed on both sides (abaxial and adaxial) with 200 µg·mL^−1^ oxytetracycline until saturation (about 5 mL). Three days later, treated leaves were collected, washed with water, and assessed for the presence of oxytetracycline. Treatments with different letters are significantly different using Tukey’s test.

The above findings indicate that leaf cuticle is probably the main barrier for foliar uptake of oxytetracycline. Thus, we conducted another experiment in the greenhouse using foliar application (spraying on both sides of leaves with 5 mL of 200 µg·mL^−1^) and leaf soaking (enclose single intact leaves in a plastic bag with 5 mL of 200 µg·mL^−1^) with oxytetracycline in the absence of adjuvants (Figure 3). Three days after treatment, only trace amounts of oxytetracycline (0.33 ± 0.01 µg·g^−1^) were detected in leaves that were sprayed on both sides, whereas three days soaking in oxytetracycline solution (200 µg·mL^−1^) resulted in higher level (12.97 ± 0.18 µg·g^−1^) (Figure 3). The soaking likely gave the oxytetracycline solution a greater duration of contact to enter though the stomata and other openings.

### 2.4. Characterization of Citrus Leaf Cuticle

**Figure 4:**
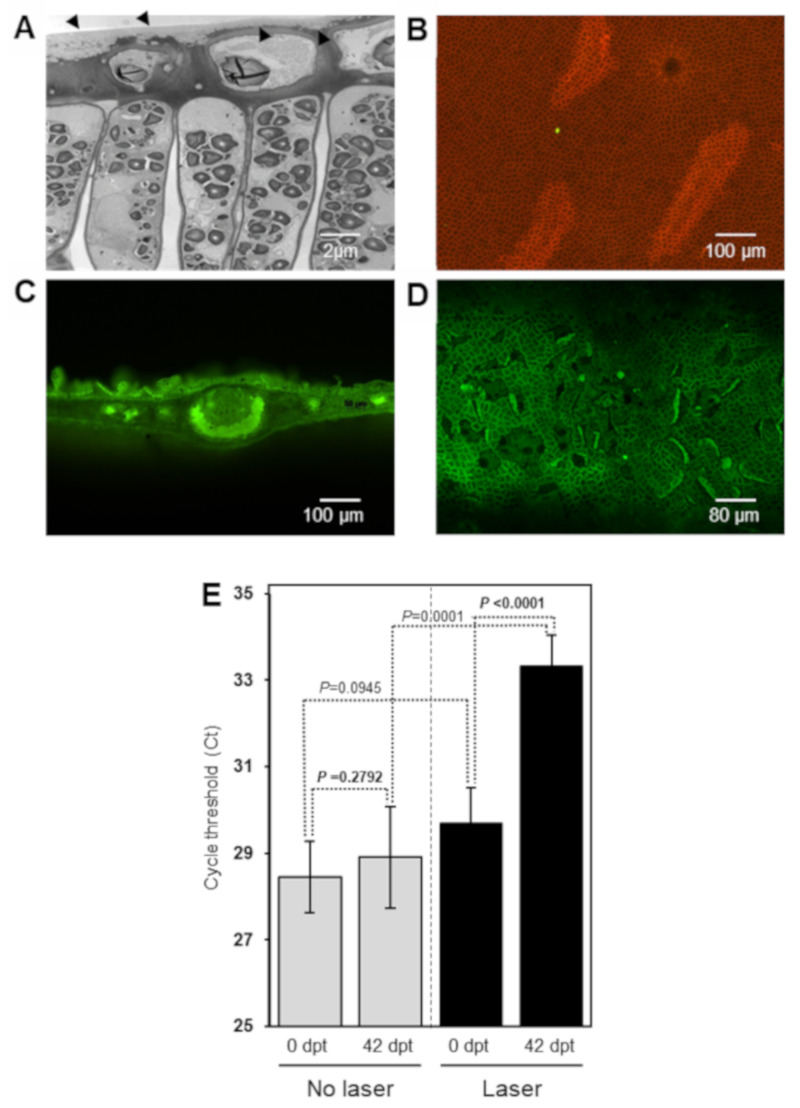
Citrus leaf cuticle is the main barrier for the uptake of oxytetracycline. (**A**) Transmission electron micrograph showing a thick cuticle layer extending between the epidermal cells (‘*Ca.* L. asiaticus’-infected leaves). (**B**) Light micrograph of isolated citrus leaf cuticle (adaxial) stained with Sudan dye showing very condensed structure. (**C**) Fluorescence micrographs of laser-perforated surface of citrus leaves (adaxial cuticle) four hours after spraying with dye (2-NDPG) for better visualization. (**D**) Fluorescence micrographs of laser-perforated leaves (section of midrib with the leaf blade) four hours after dye (2-NDPG) application. (**E**) ‘*Ca.* L. asiaticus’ titer (cycle threshold value; Ct) in laser-treated and non-treated leaves from ‘*Ca.* L. asiaticus’-infected trees at 0 and 42 d after treatment with oxytetracycline foliar spray (1000 µg ml^-1^ oxytetracycline, plus 0.25 % of commercial surfactant Tactic) (*n = 6*). The Ct at 0 and 42 day-post-treatment (dpt) were compared using two-tail *t*-test (*p* < 0.05).

The micrographs obtained from the transmission electron microscopy for the HLB-infected citrus leaves showed that the adaxial side of the leaf is protected by a thick cuticle varying from 0.5 to 1.8 µm (Figure 4A). The subtending epidermis consists of a single layer of cells with cuticular material extending into the tangential cell walls (Figure 4A). The isolated cuticle of the adaxial side is uniform and the epidermis has no stomata. The cuticle cells are very compact which may indicate the permeability to oxytetracycline is low (Figure 4B). However, perforation of citrus leaves with laser light dissolved the leaf cuticle and created openings in the leaf cuticle which allowed an increase the uptake of oxytetracycline in citrus leaves (Figure 4C and D).

### 2.5. Perforation of Citrus Leaf Cuticle with Laser Light Improves the Uptake of Oxytetracycline, which Reduces ‘Ca. L. asiaticus’ Titer (Field Study)

To test whether the leaf cuticle is the main barrier for the uptake of oxytetracycline by foliar application, we used laser light technology to create fissures on the cuticle. Foliar application of oxytetracycline to intact non-lasered citrus leaves did not affect the ‘*Ca*. L. asiaticus’ titer (Figure 4E), indicating that trace amounts of oxytetracycline were taken up by intact surface leaves. In contrast, foliar application of oxytetracycline to laser-perforated citrus leaves reduced the ‘*Ca*. L. asiaticus’ titer significantly as higher Ct values were obtained in the quantitative PCR. The *t*-test also showed that the level of ‘*Ca*. Liberibacter asiaticus’ titer after application of oxytetracycline in laser-treated leaves was significantly lower than the control (not treated with laser). The findings indicate that the cuticle is the first and main barrier for the uptake of oxytetracycline.

## 3. Discussion

Our current results showed that the adaxial side of the citrus leaf was covered by a thick lipidized cuticle. This result is in agreement with previous results showing that citrus leaves were covered with a thick cuticle [29,30]. The citrus leaf cuticle of oranges and lemons contains about 20–31 mg of wax/cm^2^ and this range is consistent during leaf development except in the juvenile stage [30]. Hydrocarbons, primary alcohols, and fatty acids are the main components of the wax of mature citrus leaves [30]. The main hydrocarbons in citrus leaf wax are *n*-entriacontane and *n*-tritriacontane, whereas *n*-hexacosanol and *n*-octacosonal are the alcohols [30]. The main components of leaf blade wax of Citrus aurantium L. were *n*-alkyl esters (C36 to C56), 1-alkanols (C24 to C40), *n*-alkanoic acids (C28 to C34), triterpenones, and *n*-alkanes (C22 to C40) [31]. The composition of the leaf blade wax was significantly affected by leaf age, side, and day and night temperature [31]. The chemical structure of the citrus cuticle suggested that it is water-repellent, and its thickness indicated that it forms a strong barrier around the citrus leaf.

The primary functions of plant cuticles are to prevent water loss and to protect plants from biotic and abiotic stresses [29]. The physical and chemical properties of the plant cuticle also make it a strong barrier against foliar applications [29]. Previous research showed that dewaxing the cuticle of citrus leaves significantly increased total penetration of urea through isolated citrus cuticle by 64%, whereas re-hydration of the urea deposit with water had minimal (~ 1% increase) effect on its penetration [32]. The total penetrations of urea through the citrus cuticle from fully-expanded leaves from 1 month-old (46.3%) and 12 month-old shoot (63.8%) were higher than those from 3 (30.1%) and 6 (30.1%) month-old shoots [32]. The rate of urea penetration through isolated cuticle decreased with increasing leaf age (from 3 to 7 week), and then increased at 9 weeks [33]. The increased penetration of urea in leaves from old flushes was attributed to the changes in wax composition and organization rather than its thickness [32]. The rate of urea penetration through isolated leaf cuticles of ‘Marsh’ grapefruit (*Citrus× paradisi* Macfad.) increased as temperature increased from 19 to 28 °C, and stayed constant thereafter until 38 °C [33]. The relative humidity increased urea penetration [33]. Cuticle thickness, cuticle weight per area increased with leaf maturity. The level of oxytetracycline in leaves that were soaked in oxytetracycline solution (200 µg·mL^−1^) for 3 d was higher than those treated with foliar application, indicating that hydration of citrus plant surface could increase the uptake of oxytetracycline as was suggested by previous studies with urea.

In both field and greenhouse experiments, only trace amounts of oxytetracycline were detected in citrus leaves upon foliar application of oxytetracycline. These results indicated poor uptake of oxytetracycline through intact citrus leaves. Conversely, the laser perforation of citrus leaves increased the uptake of oxytetracycline as indicated by the decrease in ‘*Ca*. L. asiaticus’ titer. In agreement with our results, application of BODIPY-FL vancomycin on intact citrus leaves resulted in minimal uptake of this antibiotic [29]. However, the uptake of BODIPY-FL vancomycin in laser-perforated leaves was 2511% higher than non–laser-perforated leaves [29]. The uptake of the fluorescent derivative of penicillin (Bocillin-FL penicillin) was also enhanced in laser-perforated leaves. The FL-penicillin was detected in cross sections of leaf blades and in the petiole [29]. Similar results were also obtained with other compounds including 2-(*N*-(7-nitrobenz-2-oxa-1,3-diazol-4-yl) amino)-2-deoxyglucose (2-NBDG), adenosine triphosphate (ATP), lysine, and trehalose [29]. Laser light scarification enhances the uptake of foliar applied substance by creating openings in the leaf surface, which provide direct entry into apoplast, and then to the vascular system [29].

Our results showed that adjuvants had little effect on oxytetracycline delivery. In addition, application of the surfactant Tactic did not improve the uptake of foliar-applied oxytetracycline in the field. These results showed that selected adjuvants did not enhance the uptake of oxytetracycline through foliar application. The relative effectiveness of adjuvants on foliar-applied agrochemicals depends on the type of applied compound, selected adjuvant, and plant species [34]. A previous study showed that mixing the silicone-based L-77 surfactant with the Kocide (Cu fungicide) suspension significantly increased the droplet’s surface area and enhanced the uptake of Cu through abaxial leaf cuticles (isolated), but not through astomatous adaxial (isolated) leaf cuticles [35]. However, petroleum spray oil and urea adjuvants did not affect the surface area of droplets or the uptake of Cu through ‘Marsh’ grapefruit leaves [35]. The previous results indicated that the adaxial leaf cuticle had lower permeability than the abaxial surface. In addition, the previous result suggested that addition of adjuvants to foliar applications do not improve uptake of applied chemicals because most sprayed materials settle on the adaxial surface of citrus leaf. The high level of oxytetracycline found in soaked leaves could have resulted from the uptake of oxytetracycline through the stomata present in the abaxial cuticle.

Trunk injection resulted in higher levels of oxytetracycline in leaves compared to foliar sprays. Our results are in agreement with earlier studies, which showed that trunk injection of oxytetracycline was more efficient than foliar application [6,8,10]. A recent study showed that the minimum concentrations of oxytetracycline required to suppress ‘*Ca*. L. asiaticus’ bacterium in citrus leaves to below the detection limit were 0.68 and 0.86 µg·g^-1^ under greenhouse and field conditions, respectively [36]. Initial inhibition of ‘*Ca*. L. asiaticus’ growth in citrus leaves were observed at 0.17 and 0.215 µg·g^−1^ oxytetracycline under greenhouse and field conditions, respectively [36]. Similar results were also reported by Vincent et al. (2019) [37]. In agreement with our results, foliar application of oxytetracycline did not result in significant reduction in ‘*Ca*. L. asiaticus’ titer [36]. Injection of 0.50 g oxytetracycline in 3-year-old Hamlin trees resulted in ∼ 0.30 µg·g^-1^ oxytetracycline in leaf tissues [36]. The residue levels of oxytetracycline in citrus fruits were 0.018 and 0.038 µg·g^-1^ after 9 months of injecting 0.25 g and 0.50 g of oxytetracycline in 3-year-old Hamlin trees [36]. Li et al. (2019) suggested further evaluation of oxytetracycline residues in citrus fruit and juice under field conditions [36]. Based on the minimum inhibitory concentration (MIC: ∼0.86 µg/g in leaf tissues) developed by Li et al. (2019) and Vincent et al. (2019), no adjuvant achieved this minimum threshold [36,37]. Convincingly, the level of oxytetracycline in leaves collected from trees that were treated using trunk injection was several folds higher than the MIC. Taken together, the previous and current results showed that trunk injection of antibiotics is more efficient than foliar application.

Antibiotics have been successfully used for the control of several plant disease for more than seventy years, however their use have been challenged by several issues including high cost, inefficiency at low doses, and development of resistance in plant- and animal- pathogenic bacteria due to improper application of antibiotics [38]. A recent study showed that oxytetracycline was taken up by carrot, pepper, and lettuce that were grown on manure-amended soil [39]. Accumulation of oxytetracycline increased the activity of the NADPH P450 reductase and glutathione-s-transferase enzyme in plants, indicating an induction of the detoxification process [39]. This result suggested that consuming produce grown on manure-amended soil could affect human health [39]. In addition, accumulation of antibiotics in agroecosystems may result in the spread of antibiotic resistant bacteria and antibiotic resistance genes in the environment [40]. Exposing pakchoi (*Brassica chinensis* L.) to cephalexin, tetracycline, and sulfamethoxazole increased the level of antibiotic-resistant endophytic bacteria [40]. In addition, several antibiotic resistance genes, were present in the pakchoi endophytic system after treatment with the previous antibiotics [40]. These results indicated possible development and spread of antibiotic resistance in vegetable endophytic systems upon antibiotic treatment [40].

In our previous study, we detected oxytetracycline in the outer stem, the bark (phloem), and inner stem (xylem), and leaves upon trunk application [17]. The level of oxytetracycline in the xylem was higher than in the phloem indicating that the xylem was the main route of transport [17]. The presence of oxytetracycline in the phloem indicated that oxytetracycline transported in the xylem could be redistributed into the phloem by bidirectional exchange [17]. Since trunk injection of oxytetracycline is more efficient than the foliar spray and soil drench application methods, and has several advantages over these two application methods [24], trunk injection would be the best method for the delivery of antibiotics into ‘*Ca*. L. asiaticus’-infected trees. Trunk injection of antibiotics could minimize the drift of antibiotics in the environment, reduce the amount of applied antibiotics, and lower the possibility for the development of antibiotic resistant pathogens.

## 4. Materials and Methods

**Figure 5:**
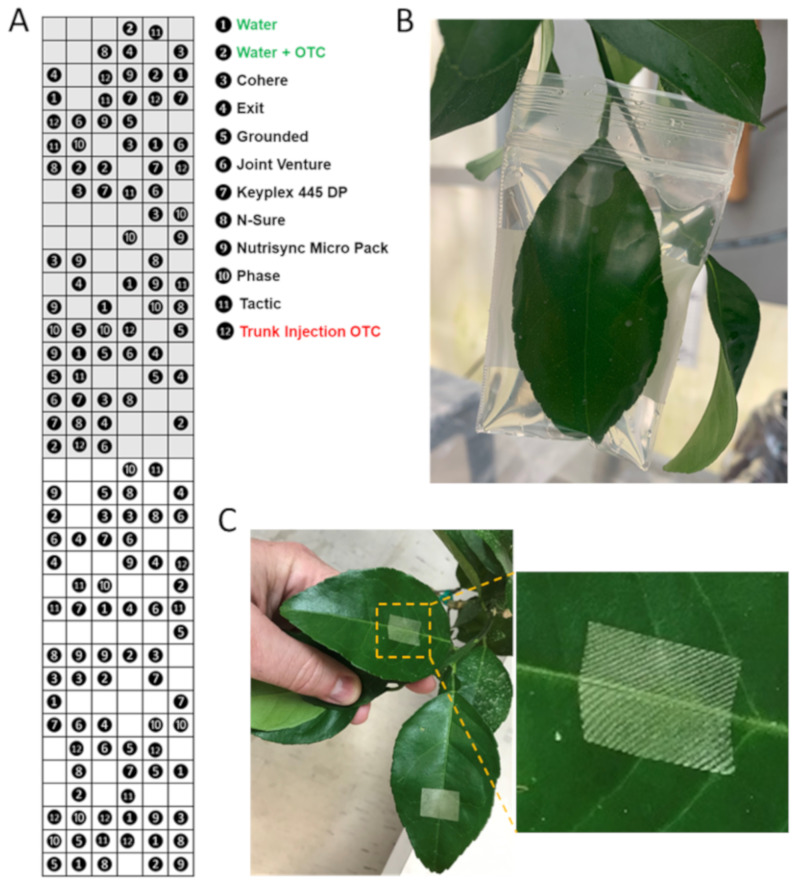
Workflow of the study. (**A**) Field experiment to assess the efficacy of different adjuvants on the foliar uptake of oxytetracycline compared to trunk injection. Twelve blocks total were included, with 2 blocks per row, and treatments were randomized within the blocks. Some trees were excluded to achieve uniform canopy size and health within each block. (**B** and **C**) Experiments to highlight the role of cuticle as a main barrier for foliar uptake of oxytetracycline. (**B**) Greenhouse experiment: leaves were bagged with excess of oxytetracycline solutions. (**C**) Field experiment: trees were treated or not with laser prior to spraying with oxytetracycline. Note the laser pattern on the leaf surface.

The workflow of this study describing the field and greenhouse experiments is shown in Figure 5.

### 4.1. Experimental Design of the Field Experiment to Assess the Efficiency of Adjuvant in the Oxytetracycline Uptake

Four-year-old trees of ‘Hamlin’ sweet orange (*Citrus sinensis* (L.) Osbeck) on Swingle citrumelo rootstock were used on November 18, 2019 for the adjuvant study. The study occurred in six rows of a planting at the Citrus Research and Education Center, University of Florida, Lake Alfred, FL (28°07′38” N 81°42′57” W). Trees were exposed to a sandy soil with a drip irrigation system. The volume of irrigation water was 0.5 gal (1.89 L) per h for 1 h daily.

The experiment was implemented in a randomized complete block design with 12 blocks and 12 treatments (Figure 5A). Blocks were arranged vertically in rows, and trees were assigned randomly to a treatment within block. Six rows were used for the experiment (Figure 5A). Each row had 2 blocks and 28 trees. The treatment structure was a factorial of 12 different treatments, and each treatment consisted of 12 trees, giving a total of 144 trees in the study (Figure 5A). Nine treatments received direct foliar application with oxytetracycline and different adjuvants (Figure 5A). In one treatment, oxytetracycline dilution was injected in the grafted part of tree (Figure 5A). One treatment was water and oxytetracycline, and one control treatment only with water applied foliarly (Figure 5A).

Three branches of each tree were flagged for leaf sampling. Before treatment, one young leaf from each labeled branch was collected for ‘*Ca.* L. asiaticus’ pre-treatment analysis. In addition, one shoot of each tree was labeled for leaf collection. Before treatment application, one shoot was bagged, and the plastic bag was removed after drying of the applied treatment. Six gallons (22.7 L) of water were used for each treatment including the control treatment. Agricultural oxytetracycline (Fireline 17 WP; 18.3% oxytetracycline hydrochloride) was obtained from AgroSource (Tequesta, FL). An amount of the agricultural oxytetracycline (110.4 g) equivalent to 18.72 g of pure oxytetracycline was dissolved in six gallons in each of the foliar spray solutions (Table 1) and was applied to 12 trees (half gallon per tree) using a CO_2_-pressurized backpack sprayer (Weed Systems Inc., Hawthorne FL). For the injection treatment, 1.56 g of Fireline 17 WP (equivalent to 265 mg of pure oxytetracycline, the same quantity of active ingredient per tree as the foliar treatments) was dissolved in 20 mL of water and injected in the trunk of selected trees using a 20-mL Chemjet manual injector (Healthy Tree PHC, Inc., Norfolk, VA, USA). Injections were made in the rootstock about 30 cm above the soil level. Four days after application, 3 leaves of each tree that received direct application (uncovered leaves) and tree leaves from shoots that were bagged (covered leaves) were collected for analysis. Twenty-eight days after application on December 20, 2019 one young leaf of the same labeled branches was collected for ‘*Ca.* L. asiaticus’ re-sampling.

### 4.2. Greenhouse Experiments

Two-year old ‘Valencia’ sweet orange (*Citrus sinensis* (L.) Osbeck) trees (84–111 cm tall) were used in these experiments (Figure 5B). Trees were maintained in a greenhouse (28  ±  1 °C, 60  ±  5% relative humidity, L16:D8 h photoperiod) at the Citrus Research and Education Center (CREC), University of Florida, Lake Alfred, USA. Five trees were used for each treatment. Three intact leaves of each plant were inserted inside a 5 × 8 cm plastic sample bag containing 8 mL of 200 µg·mL^−1^ oxytetracycline, closed with a wire twist-tie, and incubated for 3 d (Figure 5B). Alternately, three leaves from another five plants were sprayed on both sides (abaxial and adaxial) with 200 µg·mL^−1^ oxytetracycline until saturation (about 5 mL) using a spray bottle. Control plants were sprayed with deionized water or incubated with 8 mL water in bags. Three days later, treated leaves were collected, washed with cold tap water for 1 min, then washed with distilled water and dried using Kimwipes.

### 4.3. Extraction and Analysis of Oxytetracycline

Collected leaves were stored at −80 °C until analysis. The midrib was separated using a sterile razor blade and was processed for DNA extraction and PCR analysis. The remaining leaf blade was cut into small square pieces (3 × 3 mm) and about 100 mg of these pieces were transferred to 2 mL microcentrifuge tubes (a 5 mm mean diameter stainless steel ball was added to each tube). Tubes were frozen in liquid nitrogen for 10 min, placed in the sample blocks (kept frozen) and processed at 30 Hz for 1 min (2 × 30 s) using a Tissuelyzer II (Qiagen, Valencia, CA). After samples were homogenized, they were centrifuged for 1 min at 6000× *g* to bring down the leaf tissue from the cap. The homogenized tissues were mixed with 0.8 mL of 0.1 M HCl/0.01 M EDTA solution and sonicated for 30 min at room temperature, then centrifuged at 12,000 rpm for 10 min at 20 °C. The supernatant was decanted to a new centrifuge tube and the extraction procedure was repeated using another 0.8 mL extraction solution. The supernatant was collected and kept at −20 °C until analysis. Oxytetracycline ACCEL ELISA kit was purchased from Plexense, Inc., (Davis, CA, USA). The quantitation range of the oxytetracycline kit was (1.56–50 ng·mL^−1^). A 10-µL aliquot of each sample was diluted to 200 µL using the dilution buffer, which was provided with the kit, and the samples were analyzed according to manufacturer’s instructions. Briefly, 120 µL of the standard or sample was mixed with 120 µL of diluted HRP-conjugate. An aliquot (100 µL) of the mixed solution was transferred into ACCEL ELISA strip and was incubated for 30 min at room temperature. At the end of the incubation time, the mixture was discarded, and the wells were washed 6 times with the diluted washing buffer. After discarding the washing buffer, a 100 µL of the substrate solution was added and incubated for 15 min at room temperature. At the end of 15 min, the absorbance was measured using a microplate reader at 655 nm.

### 4.4. Oxytetracycline-Laser Field Experiment to Highlight the Role of Cuticle in the Uptake

Leaf surfaces of 4-year-old ‘Valencia’ sweet orange HLB-infected field trees located at the University of Florida’s Citrus Research and Education Center in Lake Alfred, Florida, were perforated with a laboratory model low-energy, CO_2_ laser-etching machine (model XY Mark-10; GPD Technologies, Peachtree City, Georgia, USA) (Figure 5C). Laser specifications used were those already reported for use in citrus fruits [29,41]. We used the dot matrix pattern where the surface area of one dot was approximately 3.14 × 10^−4^ cm^2^ and the energy per surface area of one dot = 7.85 mW dot^−1^ 10^−6^ s. Leaves were laser-perforated once on each side of the midrib. The laser-perforated area consisted of two rows of five successive rectangles. Each rectangle consisted of 15 rows of 10 perforations each (a total of 150 perforations per rectangle). The average diameter of each perforation was about 200 μm. The exposed area totaled 4.85 mm^2^ per rectangle or 48.5 mm^2^ per leaf. The depth of each perforation (pore) was about 10 epidermal cells [29].

Immediately after the laser treatment, we sprayed oxytetracycline solution on the leaf canopies of the experimental trees (6 trees per treatment). The spray solution contained 1,000 ppm (1 g·L^−1^) of the active compound, plus 0.25% of commercially available surfactant Tactic (Loveland Products, Loveland, CO, USA), the spray was applied to the adaxial side of the leaf until the solution started dripping (about 1gal per tree). Samples of two leaves from each tree were taken at time 0 and 6 weeks after the treatment, for qPCR analysis of ‘*Ca*. Liberibacter asiaticus’ using Applied Biosystems 7500 PCR instrument (Life Technologies, Carlsbad, CA, USA) as described earlier [42].

### 4.5. Cuticle Isolation

Healthy-appearing, fully expanded mature leaves from ‘Valencia’ orange (*Citrus sinensis* (L.) Osbeck) trees were used for isolation of cuticles. Leaf disks of approximately 2 cm^2^ were removed from midlamina area of leaves avoiding major veins, cuticles were isolated from leaf by digestion in an enzymatic solution containing pectinase (5% *w/v*) from *Aspergillus niger*, (CALBIOCHEM, Germany), cellulase (Onozuka RS, 0.5% *w/v*) from *Trichoderma viride*; SERVA Electrophoresis GmbH, Germany), and 50 mM Na-citrate buffer pH 4.0. To prevent any contamination, 1 mM of sodium azide was included in the solution, vacuum was applied to facilitate infiltration of enzymes into the leaf tissue, and incubation time at 35 °C was between 24–48 h. [35,43], with a few modifications.

After digestion, cuticles were separated from tissue residue by rinsing with running distilled water. For better observation of adaxial leaf cuticles, we used Sudan red 7B 0.05% solution for staining of the cuticle. High-magnification pictures of red-stained cuticle samples were taken with a Carl Zeiss AxioScope A1 fluorescent microscope (Carl Zeiss Microscopy GmbH, Göttingen, Germany) equipped with a Zeiss Axio Cam ICc1.

### 4.6. Light Microscopy (LM) and Transmission Electron Microscopy (TEM)

Leaves from experimental field trees of ‘Valencia’ orange were collected, and the samples were fixed in 3% glutaraldehyde in 0.1 M potassium phosphate buffer, pH 7.2, for 4 h at 25 °C, then overnight at 4 °C. Leaves were then washed in the same buffer and post-fixed 4 h at room temperature in 2% osmium tetroxide, stained with methylene blue/azure A, and post-stained in basic fuschin. Light micrographs were obtained from a Leitz Laborlux S compound microscope (Leitz, Wetzlar, Germany) with a Canon Powershot S31S digital camera (Tokyo, Japan). For TEM, the same blocks were thin sectioned (90–100 nm) with a diamond knife, collected on 200 mesh copper grids and stained with 2% aq. uranyl acetate, and post-stained with 5% lead citrate. TEM micrographs were taken with an AMT (Advanced Microscopy Techniques Corp., Danvers, MA, USA) digital camera mounted on a Morgagni 268 (FEI Company, Hillsboro, OR, USA) transmission electron microscope.

### 4.7. Fluorescence Microscopy

To visualize the perforation in citrus leaves and midribs after laser treatment, 2-(*N*-(7-nitrobenz-2-oxa-1,3-diazol-4-yl)amino)-2 deoxyglucose (2-NBDG), a fluorescent glucose, was applied to perforated areas and analyzed using a fluorescence microscope as described earlier [29].

### 4.8. ‘Ca. L. asiaticus’ Detection

For DNA extraction from citrus plants, leaf midribs were cut into small pieces using a razor blade (0.1 g fresh weight), placed into 2 mL screw cap tubes, and frozen in liquid nitrogen for 10 min. Frozen leaf tissue was processed at 30 Hz for 1 min (2 × 30 s) using a Tissuelyzer II (Qiagen, Germantown, MD, USA). The tubes of homogenized leaf tissue were removed from the sample blocks and were centrifuged for 1 min at 6000× *g*. DNA was extracted using potassium acetate buffer. Extracted DNA was adjusted to 100 ng/μL and then used for RT-qPCR amplification using TaqMan Universal PCR master mix (Life Technologies, Carlsbad, CA, USA) and degenerate genus-specific (*rpoB*) primer-probe sets [44]. Assays were performed in a 96-well plate using an Applied Biosystems QuantStudio 3 Real-Time PCR system and related supplies (Applied Biosystems, Foster City, CA, USA). Data were analyzed with the QuantStudio™ Design and Analysis Software (version 1.4.3) and expressed as the DNA amplification cycle threshold (Ct).

### 4.9. Statistical Analysis

JMP 9.0 software (SAS, Cary, NC, USA) was used for analysis of data. Analysis of variance (ANOVA) followed by post hoc pairwise comparisons using Tukey-Kramer honestly significant different test (Tukey HSD) were used to compare levels of oxytetracycline among the different treatments at 4 day-post-treatment (dpt) (*p* < 0.05). In addition, the levels of oxytetracycline in different treatments 4 dpt were compared to the control (water + antibiotic) using a two-tailed *t*-test (*p* < 0.05). Two-tailed *t*-tests were also used to compare the levels of oxytetracycline in covered leaves to that of uncovered leaves (*p* < 0.05). Tukey’s HSD was used to compare levels of ‘*Ca.* L. asiaticus’ titer in ‘*Ca.* L. asiaticus’-infected leaves at 0, 4, and 28 dpt (*p* < 0.05). Tukey’s test (*p* < 0.05) was used to compare the levels of oxytetracycline among control, sprayed, and soaked leaves treated in the greenhouse study. The levels of ‘*Ca.* L. asiaticus’ titer in laser-treated and non-treated leaves at 0 and 42 dpt were also compared using a two-tail *t*-test (*p* < 0.05).

## 5. Conclusions

Our results showed that low levels of oxytetracycline (below the minimum inhibitory concentration) were taken up by citrus leaves due to the presence of the thick waxy cuticle. The addition of adjuvants to the oxytetracycline solution did not enhance its uptake by citrus leaves. Laser perforation of citrus leaves enhanced the uptake of oxytetracycline by creating openings in the leaf surface confirming that the main barrier was the cuticle. Trunk injection of oxytetracycline was much more efficient than foliar application even after the addition of adjuvants. Increasing the dose of foliar application will not result in enhancing the uptake of oxytetracycline but will result in more environmental contamination and development of antibiotic resistance. Our results suggested that trunk injection would be the best method for both the delivery of therapeutic levels of antibiotics to ‘*Ca.* L. asiaticus’-infected citrus trees, and to prevent antibiotics from contaminating the surrounding environment.

## Figures and Tables

**Table 1:** Adjuvants used in foliar application.

Adjuvant	Active Ingredients	Source	* mL of Adjuvant per 6 Gallons of Water
N-Sure	Urea	Tessenderlo Kerley Inc. Phoenix, AZ	454.2 mL
Exit	Vegetable oil and dodecylphenyl ethoxylate	Miller Chemical and Fertilizer LLC, Hanover, PA	90.8 mL
Keyplex	Various mineral salts, humic acid, and yeast extract	Keyplex, Winter Park, FL	90.8 mL
Nutrysinc	Various mineral salts, including urea and ammonium nitrate	Loveland, Greeley, CO	27.3 mL
Phase	Methylated esters of fatty acids, alcohol ethoxylate, and polyether modified polysiloxane	Loveland, Greeley, CO	54.6 mL
Grounded	Aliphatic hydrocarbons, haxahydric alcohol, ethoxylates, fatty acids, and alkanolamides	Helena Company, Collierville, TN	15.6 mL
Cohere	Alkanolamide surfactants, alkylaryl polyethoxyethanol sulfates, and propanediol	Helena Company, Collierville, TN	56 mL
Tactic	Synthetic latex and organosilicone	Loveland, Greeley, CO	22.7 mL
Joint Venture	Polyalkyleneoxide modified organosilicones, ayl polyxyalkane ether, and aliphatic ester of fatty acids	Helena Company, Collierville, TN	227 mL

* Foliar solutions were prepared according to the manufacturer’s instructions, then 18.72 g of Fireline was added to the six gallons and sprayed on 12 trees (1.56 g per tree). For the injection treatment, 1.56 g of Fireline was dissolved in 20 mL of water and injected in each tree.
